# Pleiotropic Effects of PCSK9: Focus on Thrombosis and Haemostasis

**DOI:** 10.3390/metabo12030226

**Published:** 2022-03-04

**Authors:** Marianna Puccini, Ulf Landmesser, Ursula Rauch

**Affiliations:** 1 Charité Center 11—Department of Cardiology, Charité—University Medicine Berlin, 12203 Berlin, Germany; marianna.puccini@hotmail.com (M.P.); ulf.landmesser@charite.de (U.L.); 2 DZHK (German Centre for Cardiovascular Research), Partner Site Berlin, 10785 Berlin, Germany; 3 Berlin Institute of Health, 10178 Berlin, Germany

**Keywords:** proprotein convertase subtilisin/keying 9, thrombosis, coagulation, platelets, PCSK9 inhibitors

## Abstract

The proprotein convertase subtilisin/keying 9 (PCSK9) is a serine protease that has gained importance in recent years as a drug target, mainly due to its effect on cholesterol metabolism in promoting the degradation of the low-density lipoprotein receptor (LDLR). However, this protease may also play an important role in lipid-independent reactions, including the process of thrombogenesis. Considering this, we reviewed the effects and implications of PCSK9 on platelet function and blood coagulation. PCSK9 knockout mice exhibited reduced platelet activity and developed less agonist-induced arterial thrombi compared to the respective control animals. This is in line with known research that elevated blood levels of PCSK9 are associated with an increased platelet reactivity and total number of circulating platelets in humans. Moreover, PCSK9 also has an effect on crucial factors of the coagulation cascade, such as increasing factor VIII plasma levels, since the degradation of this blood clotting factor is promoted by the LDLR. The aforementioned pleiotropic effects of the PCSK9 are important to take into account when evaluating the clinical benefit of PCSK9 inhibitors.

## 1. Introduction

Proprotein convertase subtisilin/kexin type 9 (PCSK9) is a serine protease that plays an important role in lipid metabolism by promoting endosomal and lysosomal degradation of the low-density lipoprotein (LDL) receptor (LDLR). This leads to an elevation of plasma LDL cholesterol (LDL-C), enhancing the process of atherogenesis [1,2,3,4]. The gain of function mutations of the coding gene for PCSK9 have been associated with hypercholesterolemia, and, therefore, with a higher risk of coronary artery disease [5,6,7]. However, the levels of PCSK9 correlated with the rate of cardiovascular events independent of the LDL-C concentrations, which suggests that PCSK9 may play an important role for cardiovascular biology besides its effect on lipid metabolism [3,8,9]. Over the years, pleiotropic effects of PCSK9 beyond those on LDL-C metabolism have been postulated [10]. These comprise effects on vascular inflammation, inflammatory cell recruitment [11], and tumor growth [12]. PCSK9 is expressed not only in the liver on hepatocytes but also on a variety of different cells within the small intestine, kidney, pancreas, adipose tissue, and brain, as well as on the arterial wall cells, such as on endothelial (EC) and smooth muscles cells (SMC) [12,13,14].

Although the importance of PCSK9 as a cardiovascular risk factor has been mainly attributed to its effects on the LDL-C and, subsequently, oxidized LDL (OxLDL), which contributes to plaque formation [5], emerging evidence suggests an important impact of PCSK9 on platelet function and on coagulation factors, thereby modulating thrombus formation [5,15].

When addressing a possible effect of PCSK9 inhibition on platelet function, the question whether the inhibition of PCSK9 directly affects the process of thrombogenesis or indirectly by lowering the LDL-C and other lipoprotein levels arises. In this review, we focused on the effects of PCSK9 on platelet function and coagulation cascade and describe the possible underlying pathomechanisms. For this purpose we conducted a systematic search of literature on PubMed with the following MeSH terms: “PCSK9” AND (“platelet function” OR “thrombosis” OR “coagulation” OR “tissue factor” OR “von Willebrand Factor” OR “thrombin” OR “FVIII”). The articles obtained from the last twenty years were reviewed and the most important data were extracted and used for the review in order to describe the impact of PCSK9 inhibition on the process of thrombogenesis.

## 2. Effect of PCSK9 on the Vascular Wall in Relation to Atherothrombosis

The process of atherosclerosis usually ends in the generation of occlusive thrombi on the ruptured or eroded plaque or endothelium—a process known as atherothrombosis [16,17,18]. Platelets and elements of the coagulation cascade, alongside inflammatory alterations, are essential for this process [19,20,21]. PCSK9 plays an important role in the inflammation within the vascular-wall [2] and, as we will review shortly, can also affect both platelet function and coagulation, thereby contributing to atherothrombosis by affecting these three aforementioned elements.

In addition to its effects on cholesterol metabolism, PCSK9 has been shown to trigger pro-inflammatory alterations, independent of its effects on LDL-C [22]. PCSK9 stimulates the secretion of pro-inflammatory cytokines and chemokines from macrophages, and this process is mainly mediated by the LDLR [23]. The LDL-C uptake into monocytes, together with these pro-inflammatory changes, enhances the foam cell formation within the vascular-wall, contributing to the atherogenesis [2,24,25]. In addition to LDLR, the low-density lipoprotein receptor related protein (LRP-1) is another receptor involved in atherogenesis [26]. In a murine model, LDLR −/− mice that received a bone marrow transplantation from mice with selective deletion of LRP-1 in macrophages showed an increase in atherogenesis compared to mice that received normal bone marrow [27]. Since PCSK9 also downregulates the LRP-1 expression by promoting its degradation [28,29], this serine protease might affect the degree of inflammation in the vascular-wall via both LDLR and LRP-1 receptors.

Regarding the expression of PCSK9 in the atherosclerotic lesions, an interplay between PCSK9 expression and TLR4, which is the receptor of lipopolysaccharide (LPS) and a member of the Toll-like receptor (TLR) family, has also been observed [30]. In an apoE KO mouse model, Tang et al., found that by silencing the PCSK9 gene, the process of atherosclerotic lesion formation under a Western diet and the expression of inflammatory markers in the atherosclerotic aortas were both suppressed [31]. As a mechanism for the PCSK9 induced inflammatory response, the activation of the TLR4/NFκB pathway by PCSK9 was proposed [31]. Moreover, LPS has been recognized as a strong PCSK9 inducer via stimulation of TLR4/MyD88/NFκB signalling [32,33]. Another factor that upregulates the PCSK9 expression in the ECs and SMCs is the low shear stress conditions [32,33]. Under low shear stress conditions, the generation of reactive oxygen species (ROS) was enhanced and the presence of ROS even further increased the PCSK9 expression on the ECs and SMCs [33].

Furthermore, treatment with PSCK9 inhibitors, besides reducing the atherosclerotic plaque, also directly led to a reduction in arterial wall inflammation of the carotid artery when compared to the placebo [23]. This reduction in vascular-wall inflammation was independent from the alterations in circulating inflammatory markers [34]. Overall, the data suggest that PCSK9 plays an important role for the pathogenesis of atherothrombosis, not only by affecting platelets but also due to its pro-inflammatory effects, thereby perpetuating inflammatory stress on the vascular-wall.

## 3. Overview of the Effect of Lipoproteins on Platelets and Coagulation

Several studies have shown that hyperlipidemia is associated with hypercoagulability, and carries an increased risk of thrombosis [35]. Markers of platelet activation, such as increased mean platelet volume, positively correlate to cholesterol levels [36]. The increase in the LDL/HDL ratio, the apoB/apoA ratio, and the level of Lp(a) are all associated with an increased risk of venous thromboembolism [37,38]. Numerous studies suggest that the treatment of hyperlipidemia, especially with statins, reduced the risk of thrombosis [39,40,41]. Nevertheless, these effects may not only be due to the specific lipid lowering drug, but also to other underlying pathomechanisms [37].

Several mechanisms explain the effect of lipoproteins on platelet reactivity and hemostasis. First of all, upon exposure to oxidized low-density lipoproteins (OxLDL) or oxidized phospholipids (OxPL), platelets become activated [42] through their recognition by CD36 [43] or lectin-like oxidized LDL receptor 1 (LOX-1) [42,44,45]. Simultaneously, activated platelets contribute to the generation of OxLDL, which ends up propagating even more platelet activation and thrombus formation [45]. NOX-2 (the catalytic unit of NADHP oxidase) derived reactive oxygen species (ROS) is the enzymatic pathway via which platelets propagate the oxidation of lipoproteins [45,46]. Second, products of lipid peroxidation present in hyperlipidemia activate platelets by inducing the formation of the TLR1/TLR2 complex [47]. Third, Lp(a) has been identified as a carrier for the OxPL, which—as mentioned before—can activate platelets via the scavenger receptor CD36 [48]. However, regarding the direct effect of Lp(a) on platelet activation, the studies are controversial [49,50]. Fourth, it has been described that OxLDL also induces the expression of tissue factor (TF) in human monocytes in a TLR4-dependent way [51]. Additionally, finally, it has been demonstrated that, when exposed to myeloperoxidase oxidized LDL (MoxLDL9), endothelial cells have also shown to have less fibrinolysis capacity [52].

Another way in which lipoproteins can affect the haemostasis process is through the antithrombotic effects of HDL [53]. By binding to apoER2′ and to the scavenger receptor class B type I (SR-BI), HDL has been shown to induce pathways that reduce platelet activation [54,55]. HDL can also diminish platelet hyperreactivity by limiting platelet cholesterol overload via SR-BI [56]. Finally, HDL levels correlate inversely to plasminogen activator inhibitor-1 (PAI-1), suggesting a stimulation of an endogenous fibrinolytic process [57].

## 4. Impact of PCSK9 on Platelets and Blood Coagulation

### 4.1. Effect of PCSK9 on Platelet Structure and Function

**Figure 1:**
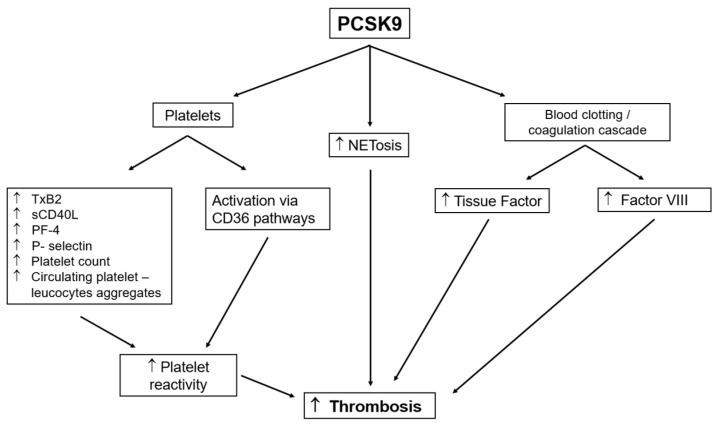
Effects of PCSK9 on platelets and coagulation cascade factors.

An association between PCSK9 levels and markers for platelet activation has been described in several studies, performed both in humans and murine models. The link between PCSK9 and platelets has been proposed to contribute to thrombogenesis (Figure 1).

In a FeCl3- induced carotid artery injury mouse model, PCSK9 −/− mice formed mainly non-occlusive unstable thrombi in comparison to PCSK9 +/+ mice. These findings suggested that PCSK9 deficiency was associated with impaired platelet function. Furthermore, platelet expression of P-selectin, levels of activated GP IIb/IIIa, and circulating platelet-leucocytes aggregates in response to injury were lower in PCSK9 −/− mice than in the non-mutant controls, pointing to a mechanism by which the loss of PCSK9 protects against coronary artery disease [58].

The PCSK9-REACT study evaluated patients with an acute coronary syndrome, who received ticagrelor or prasugrel after percutaneous coronary intervention (PCI). After quantifying PCSK9 levels and the degree of platelet reactivity by impedance aggregometry, a positive correlation was found between those two markers and the rate of major adverse cardiovascular events one year after inclusion into the study [59]. On the other hand, PCSK9 levels were also related to the cardiovascular event rate and urinary excretion of 11-dehydro-thromboxane (Tx) B2, a marker of platelet cyclooxygenase (COX)-1 activity and an indirect marker of platelet activation, in patients with atrial fibrillation [60]. These findings again reflect that PCSK9 can directly increase the rate of cardiovascular events, possibly in part due to it increasing platelet activation.

PCSK9 has been shown to enhance agonist-induced platelet aggregation [61]. Moreover, the PCSK9 expression correlates with other markers of platelet activation, such as serum TxB2 production, release of plasma soluble P-selectin, soluble CD40 ligand, platelet factor 4, platelet count, and platelet volume [61,62,63,64] (Figure 1). Furthermore, it has recently been described for the first time that platelets also express PCSK9 upon activation in the presence of LDL, and that its secretion by platelets further contributes to their aggregation and thrombus formation. In addition, this promotes monocyte differentiation into macrophages/foam cells, also contributing to atherogenesis [65].

**Figure 2:**
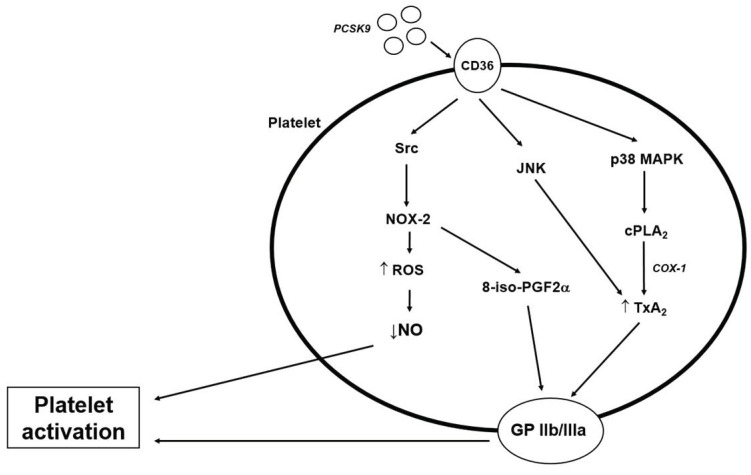
Possible effects of PCKS9 on platelets and signalling pathways that enhance platelet activation.

PCSK9 exerts its effects on platelet activation by binding to the scavenger receptor CD36 [43] and, therefore, activating CD36-dependent signalling pathways [61] (Figure 2). In patients with atrial fibrillation, Cammisoto et al., found that the interaction between PCSK9 and CD36 probably involves activation of the NOX-2, which suggests a ROS-mediating pathway. They also found that this effect is amplified by the presence of LDL [62]. Two possible pathomechanisms through which NOX-2 activates platelets have been described. First, ROS generation inactivates NO, which is a powerful antiplatelet molecule [45]. Second, NOX-2 induces the generation of platelet isoprostane 8-ISO-prostaglandin F2α (8-iso-PGF2α). This pro-aggregating molecule derived from non-enzymatic oxidation of arachidonic acid contributes to platelet recruitment via activation of GP IIb/IIIa [66].

By binding to the CD36, PCSK9 also enhances phosphorylation of Src, MAPK (mitogen-activated protein kinase)—extracellular signal-regulated kinase 5 (ERK5), MAPK-JNK (c-Jun N-terminal kinase), p38, and cytosolic phospholipase A2 (cPLA2) [61]. Src kinase becomes activated, increasing the generation of ROS by activation of NOX-2 [46], followed by activation of MAPK-ERK5 [61]. By binding to CD36, the p38 mitogen-activated protein kinase (p38MAPK) becomes active, which further cleaves cPLA2, releasing arachidonic acid from membrane phospholipids and leading to TxA2 production [5,61,62]. TxA2 then binds to its receptor and ends up activating the GP IIb/IIIa, enhancing platelet aggregation [67] (Figure 2).

### 4.2. Effect of PCSK9 on Blood Coagulation Factors

Several studies in mice and humans point to a positive association between PCSK9 and thrombotic risk [4]. Wang et al., demonstrated in a PCSK9 −/− mouse model that, after induction of venous thrombosis by partially ligating the inferior vena cava, the PCSK9 −/− mice had a reduced rate of venous thrombosis and lower length of the thrombus compared to wild mice. The PCSK9 −/− mice also showed less leucocyte accumulation and attachment and less neutrophil extracellular trap (NET) formation (NETosis) than the respective controls [68]. Since in this model the leucocyte recruitment was associated with an increased CXCL1 (a chemoattractant that enhanced NETosis [69]) expression and was P-selectin dependent, the myeloid cell recruitment is proposed as a possible mechanism by which PCSK9 enhances NETosis induced thrombosis [68]. This is consistent with the findings of Schuster et al., who showed that PCSK9 inhibition in mice decreases the expression of inflammatory chemokines, such as CXCL1 [70]. The effect of PCSK9 on NET formation is, therefore, one possible pathway through which this enzyme affects the thrombogenesis [71].

In addition, in a sepsis-mouse model, transgenic mice overexpressing PCSK9 showed increased levels of thrombin-antithrombin complexes and decreased levels of protein C, suggesting an important role for PCSK9 in the sepsis-induced hypercoagulable state [72].

When evaluating the possible relation between PCSK9 and fibrinolysis, Levine et al., found a downregulation of PCSK9 expression in mice with either pharmacologic or genetic PAI-1 inhibition. This was also observed in humans with a mutation that reduces the PAI-1 levels. Furthermore, the authors also found a positive correlation between PAI-1 and PCSK9 levels in patients with heart failure, suggesting an interplay between the fibrinolytic process and PCSK9 [73].

An elevated expression of PCSK9 was observed in patients with high antiphospholipid antibodies titers and increased thrombotic risk [74]. PCSK9 levels also correlated positively with fibrinogen levels in patients with stable coronary artery disease [75]. Overall, these data points to a positive association between the circulating PCSK9 levels and the coagulation cascade [76]. When evaluating the association of PCSK9 levels to different routine coagulation indexes in patients with angina pectoris, high PCSK9 levels correlated with low prothrombin time, and this combination predicted a poor cardiovascular outcome [77].

In patients with coronary artery disease and diabetes, the levels of PCSK9 correlate positively with those of TF [78]. TF, which is expressed in large amounts in monocytes, macrophages and in atherosclerotic plaques, contributes to thrombogenesis in many diseases [79,80] and amplifies the inflammatory response upon thrombus formation [81]. LRP-1, expressed on monocytes and hepatocytes, has been demonstrated to regulate the expression of TF by promoting its degradation [82,83]. Since PCSK9 downregulates LRP-1 expression, it might also affect circulating TF levels. Moreover, Scalise et al., described that PCSK9 can induce the TF expression on monocytes, thereby increasing the procoagulant activity. This process is mediated by the activation of the TLR4/NFκB pathway [76]. Overall, PCKS9 can increase the TF expression in both direct and indirect ways.

Another mechanism by which PCSK9 can modulate blood coagulation is through an effect on the blood clotting factor VIII (FVIII) [11,84]. Higher FVIII levels are associated with an elevated arterial and venous thrombosis risk [85,86]. LRP-1 downregulates FVIII by mediating its endocytosis and degradation [87,88,89]. Since PCSK9 has been shown to also reduce LRP-1 expression [28] and, thereby, increase FVIII levels, this is a possible explanation for how it can further contribute to thrombogenesis.

## 5. The Effect of PCSK9 Inhibitors on Platelet Function and Thrombotic Risk

The effect of PCSK9 inhibitors on platelet function has been extensively studied. Monoclonal antibodies against PCSK9 reduce platelet activation and platelet–dependent immunothrombosis [65,90]. Inclisiran, a small interfering RNA-based PSCK9, has not shown any effect on platelets thus far [91].

Marston et al., performed a post hoc analysis of the FOURIER trial and a meta-analysis of the data of the FOURIER and ODYSSEY OUTCOMES. The authors demonstrated a 31% relative risk reduction in venous thromboembolism with PCSK9 inhibition. This reduction was not related to the LDL levels but to Lp(a) baseline levels. Thus, the authors suggest the reduction in the Lp(a) levels as the underlying mechanism for this observation [92]. In contrast, other studies hypothesized other mechanisms, such as the previously mentioned effect of PCSK9 on TF and FVIII. However, these two factors were not evaluated in the aforementioned meta-analysis [84,93].

After treatment with PCSK9 inhibitors, no changes in D-dimer and fibrinogen levels were observed [94], but a reduction in plasma PAI-1 levels was documented [73], reinforcing the hypothesis of an interplay between PCSK9 and fibrinolysis.

Moreover, while on treatment with anti-PCSK9 monoclonal antibodies, platelets exhibited a decreased aggregation in the presence of aspirin. After a longer period of treatment with PCSK9 inhibition (12 months), these patients also showed decreased plasma levels of platelet activation markers, such as sCD40 L, PF-4, and soluble P-selectin, suggesting that the PSCK9 inhibition affects platelet activation beyond its lipid lowering effects [63].

Cammisotto et al., described that a possible mechanism by which PCSK9 inhibition can influence platelet activation is by downregulation of NOX-2. The reduction in the platelet activation while on treatment with PCSK9 inhibitors correlated with the decrease in OxLDL, suggesting these two pathways as possible underlying mechanisms of reducing platelet reagibility [62].

An advantage of the use of PCSK9 inhibitors as drugs that reduce platelet reactivity and thrombotic risk is that these inhibitors have not been associated with a higher bleeding risk in clinical trials thus far. In human and animal studies that evaluated the risk of hemorrhagic stroke or hemorrhagic transformation, PCSK9 inhibitors did not affect the intracerebral bleeding risk [95,96]. Nevertheless, lipid lowering therapies are often combined with very potent anti-thrombotic therapies. For example, a patient with atrial fibrillation who suffers from myocardial infarction and receives coronary stents exhibit very high cardiovascular risk, especially when a combined treatment with statin and ezetimibe fails to reduce LDL-C to levels recommended by the guidelines. Considering the described pleiotropic effects associated with PCSK9 inhibition, a simultaneous application of a PCSK9 inhibitor alongside anti-platelet drugs and anticoagulants might warrant careful monitoring for signs of bleeding, although an effect on bleeding risk by PCSK9 inhibitors has not been recognized yet.

## 6. Effect of Other Lipid-Lowering Therapies on Thrombotic Risk

As previously mentioned, hypercholesterolemia is associated with a higher risk for thrombus formation and lowering the LDL-C levels reduces this risk [97]. When evaluating the effect of other lipid-lowering therapies on thrombogenesis, there are numerous studies that suggest that statins have an effect on platelet function [98,99,100] and on thrombin formation [101], thereby reducing the risk of venous thrombosis [41]. Patients under a high intensity treatment with a statin display reduced platelet reactivity compared to patients on treatment with a statin on lower doses [102]. We previously demonstrated that when combining a low dose statin with ezetimibe in order to reach adequate LDL-C levels, the effect of the statin on platelet reactivity was diminished in comparison to the treatment with a higher dose statin alone. [98].

In addition to affecting platelet function, statins have also been shown to reduce FVIII levels [103] via the upregulation of LDLR and LRP-1 [104] and to lower von Willebrand factor levels in plasma [105], suggesting an antithrombotic effect beyond its impact on platelet activation. When comparing statins to PCKS9 inhibitors, it is important to take into account that statins have been associated with an increased risk of hemorrhagic stroke in patients with a previous hemorrhagic stroke [106].

The studies focusing on ezetimibe are controversial. Some showed that this drug can reduce platelet activation in vitro [107] and when used as monotherapy. Other studies reported no effect on platelet function when used in combination with a statin [108,109]. Contradictorily, others reported that ezetimibe lowered indices of platelet activation only when given in combination with a statin [110].

The cholesteryl ester transfer protein (CETP) inhibitors have also been investigated regarding their effect on platelet function in the past decade. CETP inhibitors are known to increase HDL-C and decrease non-HDL-C [111]. However, these drugs did not convincingly reduce the cardiovascular risk in phase III clinical trials [112]. Therefore, CETP inhibitors are not clinically available as a lipid lowering drug. Nevertheless, CETP inhibition was associated with a reduced level of platelet activation markers in studies performed on rabbits [113,114].

Overall, other lipid lowering therapies besides PCSK9 inhibitors have been found to affect the platelet function and the coagulation cascade, apparently independent to their lipid lowering effects.

## 7. Conclusions

PCSK9 affects the platelet function as well as the coagulation cascade, thereby contributing to a procoagulant and prothrombotic phenotype. The pro-thrombotic effects of PCSK9 are in part due to its impact on the LDL-C and Lp(a) levels. New evidence reveals that PCKS9 can directly regulate platelet signalling pathways and the expression of coagulation factors.

Moreover, data from clinical studies reveal that PCSK9 inhibitors lower platelet reactivity and thrombogenesis without affecting the patients’ bleeding risk thus far. More clinical studies are needed to further substantiate the safety of simultaneous application of lipid lowering drugs, such as statins and PCSK9 inhibitors, anticoagulants and anti-platelet drugs, considering the pleiotropic effects known to be associated with certain lipid lowering drugs, as discussed above.
